# Organic Vanilla Production in Mexico: Current Status, Challenges, and Perspectives

**DOI:** 10.3390/plants14243772

**Published:** 2025-12-11

**Authors:** Juan José Maldonado-Miranda, Domingo Martínez-Soto, Juan Gilberto Ceballos-Maldonado, Luis J. Castillo-Pérez, Ricardo Rodriguez-Vargas, Candy Carranza-Álvarez

**Affiliations:** 1 Facultad de Estudios Profesionales Zona Huasteca, Universidad Autónoma de San Luis Potosí, Romualdo del Campo No. 501, Rafael Curiel, Ciudad Valles 79060, San Luis Potosí, Mexico; jgilberto.cm7@gmail.com (J.G.C.-M.); jesus.perez@uaslp.mx (L.J.C.-P.); candy.carranza@uaslp.mx (C.C.-Á.); 2 Departamento de Microbiología, Centro de Investigación Científica y de Educación Superior de Ensenada, Carretera Ensenada-Tijuana No. 3918, Zona Playitas, Ensenada 22860, Baja California, Mexico; ricardo@cicese.edu.mx

**Keywords:** organic vanilla, *Vanilla planifolia*, traditional processing, vanilla production, fungal infections

## Abstract

Organic vanilla production in Mexico holds significant promise but faces substantial challenges that impact its sustainability and market competitiveness. As the native region of *Vanilla planifolia*, Mexico is endowed with rich biodiversity and a deep cultural heritage surrounding vanilla cultivation. Organic production systems in the country predominantly rely on traditional agroforestry practices, manual pollination, and artisanal curing methods, all of which enhance the quality and distinctiveness of Mexican vanilla. However, production is hindered by critical factors, including low genetic diversity and susceptibility to phytopathogenic diseases, particularly stem and root rot caused by *Fusarium oxysporum*. In recent years, the application of in vitro micropropagation techniques has shown great potential for obtaining pathogen-free plants and conserving germplasm, offering a sustainable alternative to strengthen organic systems and reduce pressure on wild populations. The labor-intensive processes, yield variability, and the complexity of adhering to organic certification standards are additional challenges to overcome. Shifts in consumer preferences toward natural and sustainably produced goods have increased demand for organic vanilla, offering Mexican producers an opportunity to gain a more prominent position in the global market. Advancing research into disease management, fostering genetic conservation, and integrating scientific advances with traditional know-how are vital strategies for overcoming current limitations. In this context, organic vanilla production represents not only an economic opportunity but also a means to conserve biodiversity, support rural communities, and maintain the legacy of one of Mexico’s most emblematic agricultural products. This review was conducted using a qualitative, narrative analysis of recent scientific literature, technical reports, and case studies related to organic vanilla production in Mexico.

## 1. Introduction

The vanilla orchid (*Vanilla* spp.) belongs to the family Orchidaceae and comprises about 110 species distributed across tropical regions of Mexico and the world; however, based on a recent revision, the genus Vanilla is now considered to include 120 accepted species plus some natural hybrids like *V. tahitensis*, reflecting newly described taxa and updated phylogenetic and morphological evidence. Unlike other orchids, vanilla is the only genus cultivated for food purposes, given that its fruit produces vanillin (4-hydroxy-3-methoxybenzaldehyde), a compound used as a flavoring in the food industry. Vanilla also holds value in medicine and perfumery. Among the species within the genus, *Vanilla planifolia*, *V. pompona*, and *V. tahitensis* are recognized globally as the main producers of natural vanillin, with *V. planifolia* contributing to nearly 95% of the world’s processed vanilla output [1,2].

*Vanilla planifolia* Jacks. ex Andrews is a hemiepiphytic plant widely distributed across the Mesoamerican region and other tropical areas of the Americas, including Mexico, Guatemala, Belize, Honduras, among others. It thrives in tropical climates under shaded to moderately lit conditions, typically at temperatures ranging from 20–30 °C [3]. Its natural habitats include evergreen and semi-evergreen tropical forests, often in karstic or rugged terrain, where it climbs host plants or trees that provide structural support, optimal light, and adequate ventilation, thereby helping reduce pest and disease incidence [4]. This broad native distribution has also contributed to significant genetic variability and a rich diversity of traditional management practices and cultivation systems associated with the species. The species typically undergoes two years of vegetative growth, with fruit production beginning in the third year. However, populations face challenges, including low density and highly dispersed distribution, which limit pollination success. Although *Vanilla planifolia* flowers are visited in their native range by male orchid bees (Euglossini), available evidence indicates that effective natural pollination rates are low and generally insufficient to support a meaningful level of fruit production. This limitation is attributed to the short lifespan of the flowers (approximately 24 h), the structural barrier separating the anther and stigma, and the absence of spontaneous self-pollination. Studies conducted across different neotropical regions, including Mexico, Guatemala, and Costa Rica, consistently report very low natural fruit set and a lack of confirmed effective pollinators, despite the presence of orchid bees and stingless bees in vanilla-bearing forests. As a result, successful fruit production in both traditional and commercial systems continues to depend almost entirely on manual pollination [4].

Despite being the center of origin and domestication of vanilla, *V. planifolia* is listed under special protection in NOM-059-SEMARNAT-2010 due to overexploitation, habitat loss, genetic erosion, and vulnerability to phytosanitary threats [5,6]. Climate change has further exacerbated declines in production. Addressing these challenges requires integrated conservation strategies and sustainable cultivation practices [7].

## 2. What Is the Organic Vanilla?

The organic flavor of vanilla comprises more than 250 compounds, with vanillin being the most abundant. This molecule is primarily extracted from the fermented pods of *V. planifolia* [8] and characterized by a clean, subtly spicy flavor profile that is difficult to replicate using conventional technological methods [9]. The classification of these products as “organic” or “artificial” depends on each country’s food regulations (Table 1).

Organic vanilla in Mexico is produced without synthetic inputs such as pesticides, herbicides, or artificial fertilizers, and must meet the requirements to be labeled and marketed as “organic.” In the country, the cultivation of vanilla under this approach began to develop in the early 1990s, along with other products such as honey, hibiscus, and avocado [10]. To be marketed in the national market, these products must comply with the provisions of the Organic Products Law (LPO) and bear the “ORGÁNICO SAGARPA MÉXICO” seal, which certifies adherence to practices that include crop management, plant nutrition, pest and disease control, as well as harvesting, transportation, processing, packaging, and labeling procedures, as well as the use of products included in the National List of Substances Allowed for Organic Agricultural Operations in Mexico; all in accordance with the principles and guidelines of organic production [11,12].

In Mexico, to obtain the “Orgánico SAGARPA México” seal, the following requirements must be met:

First, producers must implement the organic production practices established in the *Agreement that sets the Guidelines for the Organic Operation of Agricultural Activities*. Additionally, every production unit must undergo a conversion period before certification, which may range from one to three years depending on the type of production. Each producer or operator wishing to produce, certify, and market organic goods must prepare an Organic Plan that fully describes all activities carried out within the production unit. At the same time, producers must contact an Organic Certification Body (OCO) approved by SENASICA, which will guide them throughout the certification process.

Finally, once all requirements have been met, the selected OCO will conduct at least one organic inspection to verify compliance with the established procedures. If no issues are found, the appropriate certification will be issued, authorizing the use of the National Seal for organic products [13].

## 3. Traditional Processing to Obtain Organic Vanilla

Mexican organic vanilla is traditionally cured, which contributes to its high demand and richer aromatic profile [14]. Key production areas, such as the Totonacapan region and the Huasteca Potosina, adapt their methods to the local climate and resources [15]. With the goal of obtaining organic vanilla, both cultivation and curing are carried out in systems that aim to maintain productivity while avoiding or largely excluding the use of chemicals during the process, including fungicides used to prevent contamination of the final product, synthetic fertilizers, pesticides, growth promoters, or hormones [16]. This distinguishes it from conventional cultivation, where such ecological practices are generally not applied. The curing process involves four main steps: killing, sweating, drying, and conditioning. However, these stages may vary depending on climate, fruit ripeness, processing volume, and workforce availability or experience [17], making each batch of Mexican vanilla unique in quality and character.

### 3.1. Killing

This phase is the most crucial and labor-intensive step in vanilla pod curing. Its main purpose is to stop the pod’s vegetative growth, activate enzymatic processes, and delay aging [18]. This triggers the release of vanillin and other aromatic compounds by breaking down glucosyl precursors through enzymes [19]. This process damages cell membranes and walls, halting respiratory activity and causing physiological cell death. In Mexico, hot-water immersion of green beans is the predominant method [20], and some regions use scarification, ethylene exposure, or freezing [21].

### 3.2. Sweating

This phase involves storing pods in sealed, humid environments, often mahogany containers or wrapped in towels and burlap [22,23]. Enzymatic hydrolytic and oxidative reactions occur, promoting vanillin and other aroma compounds [4]. Moisture loss is controlled to inhibit microbial growth and spoilage [24]. Sweating is combined cyclically with sun drying over 5 to 30 days, enhancing enzyme activity and dehydration needed for aroma development [3]. Pods change from green to dark brown, turn more pliable in texture [25,26,27], and soften, with vanillin content reaching 60–70%, necessitating the next drying phase [28].

### 3.3. Drying and Conditioning

The curing is artisanal [29], involving oven wilting and sun drying on patios called ‘tendals’ [30]. Sun drying occurs on racks in the morning, then is shaded in the afternoon, repeated for up to three months. Over-drying can reduce flavor and vanillin content [24,31]. Finally, during the conditioning phase, the dried pods are kept at room temperature for 3 to 4 months, during which various chemical and biochemical reactions (such as esterification, etherification, and oxidative degradation) occur, enhancing the aromatic properties [32].

## 4. Production of Organic Vanilla in Mexico

### 4.1. Geographic Distribution of Organic Vanilla Production

**Figure 1:**
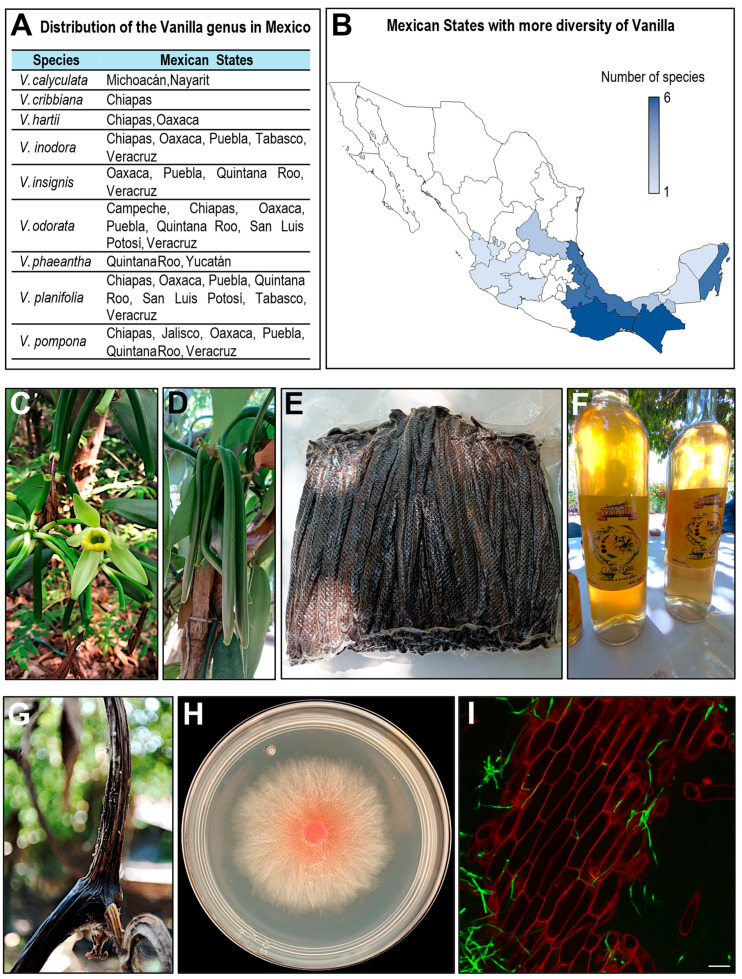
The vanilla in Mexico. (**A**) Species of the genus *Vanilla* reported in Mexico and their corresponding states. (**B**) Spatial patterns of *Vanilla* diversity in Mexico. (**C**) Flower of *Vanilla planifolia*. (**D**) Immature green pods on the vine. (**E**) Cured pods bundled for post-harvest handling. (**F**) Vanilla liquor as a commercial derivative. (**G**) Pod showing symptoms associated with *Fusarium oxysporum* f. sp. *vanillae*. (**H**) In vitro colony of the same isolate grown on solid medium. (**I**) Confocal micrography of infected vanilla tissue, showing plant tissue stained with propidium iodide (red) and fungal hyphae stained with Alexa Fluor 488 (green), scale bar = 100 μm.

Approximately 15 species have been recorded for Mexico and Central America [32]. Species recorded in Mexico are nine and include *V. cribiana*, *V. hartii*, *V. insignis*, *V. odorata*, *V. planifolia*, *V. pompona*, and *V. inodora*. In addition, taxonomic work and recent findings indicate the presence of other species, such as *V. phaeantha* and *V. calyculata* [32,33,34] (Figure 1A). In Mexico, the only species cultivated for commercial purposes is *V. planifolia*. The distribution of *Vanilla* species in Mexico exhibits a marked biogeographic pattern that reflects both the country’s ecological diversity and the environmental requirements of the genus. According to current records, *Vanilla* species occur in at least twelve states, with single-species occurrences in Jalisco, Michoacán, Nayarit, Yucatán and Campeche, and two species reported in San Luis Potosí and Tabasco. The highest richness is concentrated in the southeast, where Chiapas and Oaxaca each harbor six species, followed by Veracruz, Quintana Roo and Puebla with five species. This spatial pattern aligns with the predominance of humid tropical and subtropical ecosystems, such as evergreen and semi-evergreen forests, tropical rainforests, and cloud forests, that provide the shaded, warm and moisture-rich microhabitats required by hemiepiphytic orchids. In contrast, the western and northern regions exhibit lower species richness, consistent with their more seasonal or drier conditions, which limit suitable niches for *Vanilla* growth. Overall, species diversity increases toward the Gulf of Mexico and the southeastern region, underscoring the ecological importance of these biodiversity hotspots for the conservation, domestication and sustainable management of Vanilla genetic resources [4,7] (Figure 1B).

The “Papantla vanilla” holds a Denominación de origen, recognized as industrial property by the Mexican Institute of Industrial Property in 2011 [5,35]. This designation led to the creation of the Mexican Official Standard NOM-182-SCFI-2011 [36], which establishes the organoleptic, commercial, and testing specifications for extracts and derivatives. Its production is limited to municipalities in Veracruz and Puebla, particularly in the Totonac region, where historical, cultural, and environmental conditions support a high-quality product [5].

The high demand for vanilla in the European market caused countries such as Indonesia and Madagascar to become the largest producers of Mexican vanilla, displacing Mexican producers [19]. Furthermore, in 1874, German chemists Ferdinand Tiemann and Wilhelm Haarmann created synthetic vanilla and developed a method for its production from conifer resin. This discovery enabled us to produce vanilla in laboratories, offering a cheaper, more accessible alternative to natural vanilla extract [37].

In Mexico, organic vanilla is mainly certified under international schemes such as the United States Department of Agriculture’s National Organic Program (NOP) and the EU Organic Regulation of the European Union, which aim to regulate sustainable agricultural practices and protect biodiversity [38] (Table 1). Likewise, there are national certifying bodies, among which CERTIMEX stands out, whose objective is to strengthen the country’s agricultural, livestock, agro-industrial, and forestry production through the inspection and certification of processes and products.

The Totonacapan region, which encompasses northern Veracruz and the Sierra Nororiental of Puebla, is home to a significant number of producers offering vanilla and vanilla derivatives (Figure 1F) produced using organic practices, several of which are currently certified [39].

### 4.2. Agricultural Requirements to Produce Organic Vanilla

*V. planifolia* is cultivated under three main production systems: (1) traditional agroforestry systems, (2) semi-intensive systems, and (3) intensive or technified systems. These differ in scale, management level, suitability for organic certification, and yield potential. Vanilla production is carried out through three main systems that differ in management intensity, scale, suitability for organic certification, and productivity. Traditional agroforestry systems, which dominate organic production, rely on natural shade, living tutors, and minimal external inputs; they are typically managed by smallholders (0.25–2 ha) and yield 50–500 kg/ha of green vanilla, offering low costs and ecological benefits but limited profitability and higher vulnerability to diseases. Semi-intensive systems combine living tutors with simple support structures and improved shade and soil management, allowing small to medium farms (1–5 ha) to increase yields to 500–1500 kg/ha while remaining compatible with organic practices. In contrast, intensive or technified systems use artificial tutors, fertigation, and controlled environments to achieve the highest yields—1400–3000 kg/ha, and occasionally up to 3–4 ton/ha—but depend heavily on agro-industrial inputs, making them unsuitable for organic certification despite providing a 600–800% increase over traditional yields.

Organic production requires sustainable agricultural practices that guarantee product quality and compliance with international certification standards. Soils must be fertile, rich in organic matter, well-drained, and located in humid tropical regions with temperatures of 20–30 °C and rainfall above 1500 mm per year [40,41].

Fertility management relies exclusively on natural inputs such as compost, decomposed manure, vermicompost, or bioferments, while synthetic fertilizers, chemical pesticides, and growth regulators are prohibited [42,43]. Pest and disease control follows an integrated approach that involves plant extracts, essential oils, and beneficial microorganisms such as *Trichoderma*, along with preventive practices such as humidity control, ventilation, sanitary pruning, and removal of infected material [40,44].

A fundamental aspect of this crop is manual pollination [45], a mandatory practice in Mexico due to the lack of efficient natural pollinators [46,47]. This activity must be carried out under strict documentary records that guarantee traceability. Finally, organic certification requires a rigorous documentation and traceability system, which includes the registration of all agricultural practices, the separation of organic batches from conventional ones, and periodic verification by accredited organizations such as CERTIMEX, Ecocert, or Control Union [38].

## 5. The Market for Green and Processed Vanilla: Mexico and International Perspectives

The freshly harvested pod of *V. planifolia*, known as green vanilla (Figure 1D), is the foundation of the entire value chain. Although Mexico is the crop’s center of origin, it currently contributes less than 1% of global production yet remains highly valued for the quality and prestige of its beans, particularly those from Papantla, Veracruz [48]. In recent years, national production has ranged from 500 to 700 tons, far below leading producers such as Madagascar, Indonesia, and Uganda, but Mexican vanilla continues to occupy a niche market focused on quality and origin [48].

Organic vanilla production provides important economic advantages compared to more technologically intensive systems. While intensified cultivation can reach 1400–3000 kg/ha, and in some cases up to 3–4 tons/ha, representing a 600–800% increase over the 50–500 kg/ha typical of traditional systems, these gains require high investments in agro-industrial inputs, infrastructure, and specialized labor. Organic systems, grounded in agroforestry management and low external inputs, reduce production costs and access premium markets that value high-quality, traceable Mexican vanilla. These economic contrasts strongly shape producers’ decisions: organic production offers greater financial stability and lower dependency on costly inputs. However, traditional systems well aligned with organic certification and crucial for maintaining ecosystem services still face low profitability, erosion of traditional knowledge, and mounting pressure for land-use change, all of which threaten their long-term viability.

Production variability also drives price fluctuations: limited global supply raises prices for green and cured beans, whereas increased output depresses them, generating uncertainty for producers [49]. Despite these challenges, opportunities remain. Targeting premium niches, differentiating products by origin and production system, and strengthening producer cooperatives to guarantee traceability and stable volumes can improve competitiveness. Gourmet consumers and high-end pastry industries particularly value the cultural heritage and unique sensory qualities of Mexican vanilla, supporting premium prices for certified, single-origin batches.

### Vainilla Market

Mexico’s wholesale prices for high-quality vanilla are notably higher than those in Madagascar, with Mexican vanilla averaging USD 31,100–34,600 per ton, while Madagascar’s prices range from USD 6100 to 23,900 per ton. Indonesia can match or even exceed Mexico, with top-quality vanilla fetching up to USD 37,300 per ton, depending on bean quality and grade. These price differences highlight the significance of quality, certification, and target markets, as not all vanilla achieves the same valuation.

The processed vanilla market is experiencing robust growth, with projected compound annual growth rates of 5–8% for products such as pastes and extracts. This expansion is driven by increasing demand in gourmet foods, bakery products, ice cream, and health-oriented beverages.

Regulatory frameworks are crucial in this context. In Mexico, the Organic Products Law [50] establishes the requirements for labeling and certification of organic vanilla, while exports must also comply with international standards, including USDA Organic [51] and EU regulations.

## 6. Phytopathogenic Threats of Vanilla Cultivation and Emerging Control Strategies Against *Fusarium oxysporum*

Cultivated vanilla has been extensively cloned, resulting in reduced genetic diversity among cultivars worldwide [52,53]. This lack of diversity limits their adaptability to adverse environmental conditions and biotic stresses, such as diseases caused by phytopathogens. Globally, vanilla crops have suffered from serious phytosanitary issues due to various pathogenic microorganisms. For instance, viral diseases like Cymbidium mosaic virus, potyvirus, and cucumber mosaic virus cause leaf mosaic and deformation [54]. Additionally, soft rot symptoms caused by *Dickeya dadantii* (such as water-soaked brown spots on leaves and stems, brown margins, and oozing white exudate) have been reported in vanilla plantations in China [55].

In addition to viral and bacterial diseases, a range of fungal pathogens significantly impact vanilla crops. Comprehensive surveys in Mexican vanilla-growing regions have documented numerous pathogenic species (Table 2). Anthracnose, caused by Colletotrichum species including *C. gloeosporioides*, has been reported in Veracruz [56]. Rust diseases, resulting from *Uromyces joffrini* and *Puccinia sinanoemea*, are also present in Veracruz plantations [57]. *Lasiodiplodia theobromae* causes rotting and dieback, while *Neopestalotiopsis rosae* is associated with leaf blight and plant rot [56]. Other documented diseases include canker by *F. pseudocircinatum*, white rot by *Sclerotium* sp. [56], and widespread infections by *Fusarium proliferatum* and *F. solani* [56,58] (Table 2).

Globally, the most devastating diseases for vanilla production are root rot, caused by the cosmopolitan fungus *Fusarium oxysporum*, and basal rot of stems and roots, caused by *Phytophthora* spp. [57,59] (Table 2). In Mexico, *F. oxysporum* f. sp. *vanillae* stands out as the most destructive pathogen, responsible for Vanilla wilt, a disease resulting in severe plantation losses and significantly diminished vanilla yields. This pathogen has been extensively reported in several states, affecting *V. planifolia* in municipalities across Veracruz [57,58,60,61], as well as *V. planifolia* in San Luis Potosí and *V. pompona* in Nayarit [60,61] (Table 2).

**Table 2:** Pathogenic fungi affecting vanilla crops in Mexico.

Fungi	Disease	*Vanilla* Specie	Municipalities	State	References
*Colletotrichum* sp.	Anthracnose	*V. planifolia*	-	Veracruz	[62]
*Uromyces joffrini*	Rust	*V. planifolia*	-	Veracruz	[62]
*F. oxysporum* f. sp. *vanillae*	Vanilla wilt or vanilla root rot	*V. planifolia*	Papantla	Veracruz	[58]
*F. proliferatum*	Widespread plant diseases	*V. planifolia*	Papantla	Veracruz	[58]
*Fusarium* sp.	Vanilla wilt or vanilla root rot	*V. planifolia*	Papantla	Veracruz	[58]
*F. oxysporum*	Vanilla wilt or vanilla root rot	*V. pompona*	Xalisco, Ruiz	Nayarit	[61]
*F. oxysporum*	Vanilla wilt or vanilla root rot	*V. planifolia*	Matlapa	San Luis Potosí	[60]
*Fusarium* spp.	Vanilla wilt or vanilla root rot	*V. planifolia*	Papantla	Veracruz	[63]
*Colletotrichum gloeosporioides*	Anthracnose	*V. planifolia*	Papantla	Veracruz	[56]
*Fusarium solani*	Widespread plant diseases	*V. planifolia*	Papantla	Veracruz	[56]
*Lasiodiplodia theobromae*	Rotting and dieback	*V. planifolia*	Papantla	Veracruz	[56]
*Neopestalotiopsis rosae*	Leaf blight and plant rot	*V. planifolia*	Papantla	Veracruz	[56]
*F. pseudocircinatum*	Canker	*V. planifolia*	Papantla	Veracruz	[56]
*F. oxysporum*	Vanilla wilt or vanilla root rot	*V. planifolia*	Papantla, Gutiérrez Zamora, Actopan, Vega de Alatorre, San Rafael, Tlapacoyan.	Veracruz	[64]
*F. oxysporum* f. sp. *vanillae*	Vanilla wilt or vanilla root rot	*V. planifolia*	Emilliano Zapata	Veracruz	[57]
*Puccinia sinanoemea*	Rust	*V. planifolia*	Emilliano Zapata	Veracruz	[57]
*F. oxysporum* f. sp. *vanillae*	Vanilla wilt or vanilla root rot	*V. planifolia*	Papantla	Veracruz	[65]

### 6.1. F. oxysporum f. sp. Vanillae as Main Treat of Vanilla

*F. oxysporum* f. sp. *vanillae*, previously known as *F. batatis* Wr. var. *vanillae* causes chlorotic rings on the stems and basal rot, general wilting of the plants, tissue dries out, blackening or necrosis (Figure 1G), and finally the death of plants [66]. So far, in Mexico, this disease has been comprehensively documented across vanilla-producing regions, representing the most significant threat to vanilla cultivation in the country (Table 2).

A particular case is that the situation in Huasteca Potosina faces significant constraints due to the interaction of biophysical and socio-economic factors that affect productivity and crop resilience [41]. Phytosanitary issues are among the most critical challenges, especially infections caused by *Fusarium oxysporum*, *Phytophthora* spp., and other opportunistic pathogens, which are frequently reported in the region [52,53]. These diseases severely affect the roots, stems, and pods of *Vanilla planifolia*, frequently leading to plant decline and losses in pod yield and quality.

Environmental pressures in the Huasteca Potosina, such as deforestation of tropical forests, soil erosion, and the increasing variability of rainfall and temperature, exacerbate pest and disease incidence [41,42]. Irregular climatic patterns disrupt flowering induction and fruit setting, key processes in vanilla productivity.

In addition, socio-economic conditions, including the marginalization of rural communities, limited access to technical training, and low market integration, reduce the competitiveness of local production systems. This scenario reinforces dependence on intermediaries and restricts producers’ income generation. Security problems in vanilla plantations (particularly pod theft) often lead to premature harvesting, hindering proper biochemical maturation and reducing the product’s commercial value.

Overall, these findings highlight the urgent need for integrated management approaches in the Huasteca Potosina, combining phytosanitary improvements, agroforestry diversification, and strengthening social and commercial structures to ensure the sustainability and profitability of vanilla cultivation in the region (Table 3).

Knowledge of *F. oxysporum* f. sp. *vanillae* and its interactions with the host remains limited, offering a significant opportunity to understand its pathogenesis and control its spread. In-depth research on the molecular basis of pathogenicity, host resistance, and environmental factors is crucial. Integrating genomic, transcriptomic, and metabolomic approaches could inform effective management strategies, including early detection, the development of resistant cultivars, and biological control options to support the sustainable production of *V. planifolia*.

### 6.2. Emerging Management Strategies Against F. oxysporum

Research on alternatives for the management of fungal diseases in agriculture, driven by the growing challenge of fungicide resistance, has increasingly focused on the exploitation of natural metabolites. Plant essential oils, such as those from *Citrus sinensis*, exhibit high inhibitory activity against *F. oxysporum* by targeting polyketide synthase beta-ketoacyl synthase domain; in silico analyses identified nootkatone as a lead compound with strong binding affinity [67]. The extracts of *Larrea tridentata* (gobernadora) efficiently inhibited *F. oxysporum* f. sp. *radicis-lycopersici* under in vitro and in vivo conditions in greenhouse trials, with dichloromethane and methanol extracts showing the highest inhibition percentages, although the precise mechanism of action remains unknown [68].

Another novel approach for the management is the green-synthesized nanoparticles, which represent a sustainable alternative for the control of phytopathogens like *F. oxysporum*. These particles, synthesized using biological materials such as plant extracts or bacterial filtrates [69], demonstrate greater antifungal efficacy than chemically synthesized counterparts and require lower concentrations for in vitro inhibition of *Fusarium* growth [67]. Selenium nanoparticles (SeNPs), copper nanoparticles (CuNPs), and zinc oxide nanoparticles (ZnONPs) have all shown dose- and time-dependent activity against *F. oxysporum* across multiple species [70,71,72]. Notably, nanoparticle size significantly influences effectiveness, for example, AgNPs of 2 nm diameter demonstrated superior antifungal activity compared to larger variants in another *Fusarium* species [73]. Synergistic approaches combining metal nanoparticles with beneficial fungi, such as *Trichoderma harzianum*, have yielded significant reductions in Fusarium wilt while promoting plant growth under both greenhouse and field conditions of economic important crops [74], suggesting potential applicability for managing Fusarium in vanilla cultivation.

### 6.3. Development of Resistant Vanilla Cultivars for Management of Fusarium oxysporum f. sp. Vanillae

The development of resistant vanilla cultivars represents a sustainable long-term strategy for managing Fusarium wilt. The main cultivated species, Vanilla planifolia, is generally highly susceptible to *F. oxysporum* f. sp. *vanillae* (Fov) and *F. oxysporum* f. sp. *radicis-vanillae* (Forv), which cause vascular wilt and root-stem rot, respectively [66,75].

Wild *Vanilla* species, particularly *V. pompona*, exhibit robust natural resistance to *F. oxysporum* f. sp. *vanillae* (Fov) and *F. oxysporum* f. sp. *radicis-vanillae* (Forv) [63,75]. Experimental crosses between *V. planifolia* and *V. pompona* have produced hybrids that inherit resistance traits regardless of which species serve as the pollen donor or receptor. Barreda-Castillo et al. [75] demonstrated that *V. planifolia* × *V. pompona* hybrids showed only 9.6–14.8% root damage when challenged with Fov at 30 °C, compared to 100% damage in pure *V. planifolia*. These hybrids were classified as “highly resistant to slightly resistant,” confirming that resistance is heritable. Similarly, the triple hybrid *Vanilla* cv. Vaitsy (*V. planifolia* × *V. pompona* × *V. planifolia*) has been reported to be highly resistant, producing idioblasts in the roots that restrict pathogen progression [75,76]. Additional resistant species include *V. phaeantha*, *V. bahiana*, *V. crenulata*, and *V. costariciensis* [77].

## 7. In Vitro Micropropagation: A Strategy for the Conservation and Production of Organic Vanilla

The commercially valuable orchid *V. planifolia* faces several agronomic and phytosanitary challenges, including low seed germination under natural conditions, reliance on hand pollination, and a high incidence of pathogenic microorganisms [20]. Given these limitations, plant biotechnology has become a strategic tool for producing pathogen-free plants, ex situ conservation of germplasm, and mass propagation of plants for organic and sustainable farming systems [78].

In vitro micropropagation protocols for *V. planifolia* have incorporated several techniques and methodologies, such as symbiotic and asymbiotic seed germination [79,80,81,82], shoot tip and node culture [83], direct and indirect organogenesis [84,85], and the production of synthetic seeds and protocorm-like bodies (PLB’s) for conservation and propagation purposes [83,86]. The most commonly used culture media include Murashige and Skoog (MS) and its variations, which may be diluted or supplemented with organic extracts (e.g., coconut water or pineapple and apple extracts). In addition, plant growth regulators, primarily cytokinins and auxins, are used to stimulate shoot induction, elongation, and rooting [87,88,89,90].

Among these techniques, asymbiotic seed germination has been widely used to obtain seedlings from capsules, using protocols that consider the seed maturity stage, disinfection treatments, and the composition of the culture medium [78,81], particularly regarding sugar concentration and photoperiod [88]. On the other hand, the harvest stage and the embryo’s physiological state are determining factors for the success of in vitro germination, underscoring the need to adapt protocols to the physiological conditions of the plant material used [91].

**Figure 2:**
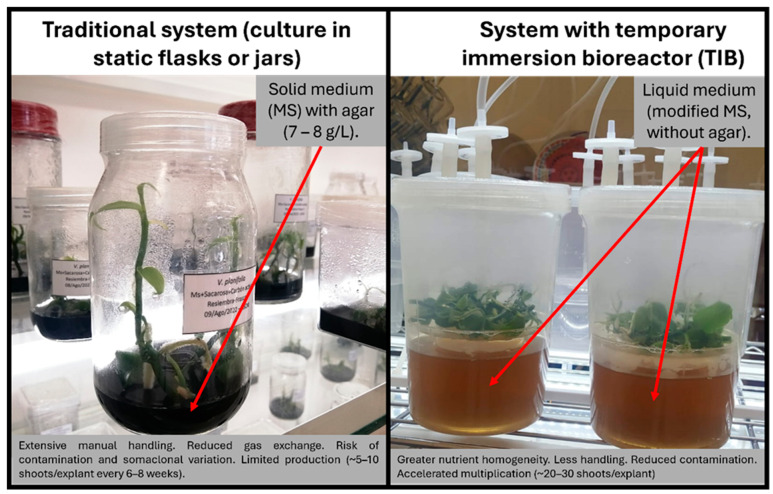
Comparison between the conventional micropropagation system (static culture in solid medium) and the temporary immersion bioreactor (TIB) system applied to *Vanilla planifolia*. TIBs allow intermittent contact of the explant with the liquid medium, improving aeration, nutritional homogeneity, and multiplication efficiency, as well as reducing production costs and microbial contamination.

To increase efficiency and reduce production costs, temporary immersion bioreactor (TIB) systems have been developed. These systems have demonstrated significant advantages over traditional methods, as they improve aeration and nutrient exchange, reduce microbial contamination, and enable automation of the multiplication process (Figure 2). As a result, TIBs are considered a viable alternative for the large-scale production of in vitro plants in bioreactors, and several recent studies report notable increases in the multiplication rates of *V. planifolia* under this system [92,93,94,95].

In Mexico, advances in vanilla micropropagation and conservation protocols have developed considerably in recent decades. Several universities and research institutes such as the Universidad Veracruzana, through its Center for Tropical Research, the National Institute of Forestry, Agricultural and Livestock Research (INIFAP), the Center for Scientific Research of Yucatán (CICY), the Autonomous University of San Luis Potosí, and others regional universities have developed protocols and technical improvements focused on increasing the efficiency of germination, multiplication, and acclimatization of vitroplants [90,95,96,97,98,99,100]. Among the main Mexican contributions are the optimization of culture media, the use of natural organic extracts to reduce dependence on synthetic regulators, and the implementation of conservation methods under controlled osmotic stress conditions.

The use of organic compounds, such as chitosan and extracts from various fruits, has been shown to stimulate morphogenesis and rooting, while controlled osmotic treatments have favored germplasm conservation [85,90]. These advances represent viable alternatives for producing certified plant material in organic systems, where the absence of chemical and phytosanitary contaminants is fundamental.

The micropropagation process plays an essential role in producing plant material that meets organic certification criteria. This technique not only allows for obtaining plantlets free of chemical residues and pathogens but also facilitates the conservation of genetic diversity through the creation of in vitro germplasm banks and the production of PLB’s or synthetic seeds [78,83,91]. In Mexican regions with a vanilla-growing tradition, such as the Totonacapan in Veracruz and the producing areas of Puebla and Oaxaca, the availability of healthy and genetically homogeneous material contributes to strengthening production chains and improving productivity without compromising ecological standards [37,61,64].

However, despite its biotechnological potential, the practical adoption of in vitro propagation in rural Mexican vanilla-growing communities remains limited. Many smallholder producers face substantial barriers, including restricted access to laboratory facilities, limited training in plant tissue culture, high initial investment costs, and skepticism toward externally developed technologies that reduce the feasibility of integrating micropropagated plants into traditional agroforestry systems [41]. Furthermore, the lack of locally adapted protocols for diverse native genotypes may lead to poor field performance, undermining producer confidence in these methods. These socio-economic and technical constraints highlight that micropropagation, while promising, cannot be assumed to be universally accessible or immediately scalable in rural contexts without coordinated extension programs, long-term support, and participatory technology transfer [96,98].

Despite the progress achieved, several challenges remain for the widespread adoption of these technologies among producers. These include limited technology transfer, the need for technical training, initial implementation costs, adapting in vitro plants to the shaded conditions of traditional agroecosystems, and the lack of specific protocols for different local genotypes [41,44,49,78]. Future research should focus on standardizing cost-effective protocols (including the commercial-scale use of in vitro plant propagation), evaluating the agronomic and phytosanitary performance of micro-propagated plants under organic management, and establishing networks for the conservation and distribution of virus- and fungus-free material that maintains regional genetic diversity.

The accumulated international evidence, along with relevant contributions from Mexico, demonstrates that in vitro micropropagation is a tool with the potential to provide scalable solutions compatible with organic agriculture [42,43]. Integrating these biotechnological strategies with agricultural extension programs and organic certification schemes is among the most promising ways to strengthen local value chains and safeguard vanilla’s genetic heritage.

## 8. Perspectives of Organic Vanilla Production

Organic vanilla production is a growing yet demanding sector within the global flavor and fragrance industry. As consumers increasingly seek natural and sustainably sourced ingredients, organic vanilla has gained prominence for its quality, health benefits, and environmental attributes. In Mexico, the native range of *V. planifolia* provides a unique opportunity to recover part of the country’s historical leadership while promoting biodiversity conservation and supporting smallholder livelihoods [101]. Agronomically, organic cultivation fosters environmentally friendly practices such as the use of natural fertilizers, integrated pest management, and the exclusion of synthetic agrochemicals, which improve soil health, ecosystem stability, and the organoleptic quality of cured beans, facilitating access to premium markets. However, the sector continues to face structural challenges, including lower yields, higher labor requirements, vulnerability to pests and diseases, and the complexity of post-harvest processing. In key producing states such as Veracruz and San Luis Potosí, infections caused by *Fusarium* spp. remains one of the primary constraints, particularly in traditional and rustic production systems [63].

A critical additional dimension is the socioeconomic context in which vanilla cultivation occurs in Mexico. Production is largely carried out by smallholders managing plots of less than 1 ha, for whom vanilla is often a complementary rather than primary economic activity. This configuration makes the crop especially vulnerable to shifts toward more profitable or less labor-intensive alternatives, most notably citrus crops, which have expanded considerably in major vanilla-growing regions such as Veracruz and San Luis Potosí. The future viability of organic vanilla production must therefore be examined in relation to these competing land-use trends, assessing both market incentives and producers’ capacity to sustain long-term investments in agroforestry-based vanilla systems amid increasing pressure from alternative commodities.

In addition, the expansion of organic vanilla production is increasingly shaped by national agricultural policies and rural development programs. Initiatives such as “Sembrando Vida”, which promote agroforestry diversification and ecological restoration, offer strategic opportunities for strengthening sustainable vanilla cultivation. The program’s emphasis on shaded polyculture systems, incorporation of native tree species, and direct support for smallholder farmers aligns with the ecological requirements of *V. planifolia* and the socio-productive structure of traditional vanilla regions. These policy frameworks could significantly enhance the resilience of vanilla agroecosystems by improving access to technical assistance, fostering landscape-level biodiversity, and creating more stable income opportunities. Nevertheless, capitalizing on such policy-driven advantages will require the effective integration of vanilla into existing agroforestry models, long-term producer training, and the alignment of local market incentives with national sustainability objectives.

## 9. Conclusions

Organic production of *V. planifolia* in Mexico represents a strategic opportunity both economically and environmentally, given the growing value of organic vanilla in international markets and its status as a native crop of the country. However, the reviewed data indicate that this activity faces significant challenges, including low yields, high vulnerability to pests and diseases, limited availability of skilled labor, and the complexity of organic certification processes. Despite these limitations, implementing sustainable agricultural practices, improving disease management, particularly of wilt caused by *F. oxysporum*, and strengthening value chains can enhance both productivity and profitability. The conservation of genetic diversity, along with the integration of traditional and scientific knowledge, is essential to ensure the long-term sustainability of the crop. Looking ahead, Mexico has the potential to become a leading supplier of high-quality organic vanilla, while simultaneously contributing to rural development, biodiversity conservation, and competitiveness in high-value markets.

## Figures and Tables

**Table 1:** International organizations that certificate organic vanilla.

Certification Name	What Is It?	How Does It Apply to Mexican Vanilla?
LPO (Organic Products Law, Mexico)	National regulations governing the production and marketing of organic products in Mexico. Includes the “Organic Mexico” label.	It is mandatory to market vanilla as organic within Mexico. Producers must obtain certification from organizations accredited by the National Service of Health, Safety and Agri-Food Quality (SENASICA).
USDA NOP (National Organic Program, EE. UU.)	Regulation of the United States Department of Agriculture that defines the requirements for labeling products as “USDA Organic”.	Mexican producers can obtain certification from accredited agents to export organic vanilla to the U.S. market.
EU Organic (European Union Regulation)	European Union regulations governing organic production and the use of the “EU Organic” logo.	Mexican exporters must have certification issued by an EU-recognized body to import vanilla into the European market.
JAS (Japanese Agricultural Standards)	Official system of Japan that regulates organic production standards in that country.	Mexican producers can obtain JAS certification through accredited organizations, allowing them to export organic vanilla to the Japanese market.
Canada Organic Regime (COR)	Canada’s regulatory framework for organic product certification, managed by the Canadian Food Inspection Agency.	Mexican producers can certify their vanilla under COR or export through equivalence agreements with other international certifications.
Other national regimes (i.e., Korea, China GB/T)	Country-specific regulations governing the production, certification, and labeling of organic products.	Mexican vanilla can access these markets if it meets local certifications or through equivalence agreements with accredited certifiers.

**Table 3:** Main constraints to vanilla production.

**Category**	**Main Causes**	**Consequences**	**Observations**
Phytosanitary	Fungal infections (*Fusarium oxysporum*, *Phytophthora* spp.). Inadequate agronomic management. Poor hygienic practices during curing.	Root and stem rot Reduced yield and plant mortality Pod deterioration (spots, unpleasant odor, loss of aroma).	Fungal pathogens identified by the Environmental Sciences Research Laboratory (FEPZH-UASLP).
Environmental	Deforestation (up to 80% loss of forest cover). Climate change (temperature and rainfall alterations). Unsuitable land use and lack of agroforestry practices.	Altered flowering and fruit set. Soil erosion and reduced fertility. Increased incidence of pests and diseases.	Extreme humidity conditions promote infections.
Social	Marginalization and poverty in producing communities. Limited access to financial support and credits. Low producer organization.	Dependence on intermediaries. Low income Economic and social inequality.	Farmers often sell pods at very low prices.
Economic	Limited added value in the final product. Lack of technical and commercial training. Unequal market competition.	Low national and international competitiveness. Significant economic losses.	Income reduction is associated with poor product quality.
Insecurity and vulnerability	Theft of vanilla pods Labor migration and aging producer population.	Premature harvesting to prevent losses. Reduced product quality. Reduced local labor availability.	Robberies are frequently reported during pod maturation season.

## Data Availability

No new data were created or analyzed in this study.
